# Monitoring and evaluation of hypotension in the extremely preterm

**DOI:** 10.3389/fcvm.2024.1477337

**Published:** 2024-10-02

**Authors:** Ping Ping, Beimeng Yu, Renjie Xu, Pingping Zhao, Shuqi He

**Affiliations:** Shaoxing Maternity and Child Health Care Hospital, Maternity and Child Health Care Affiliated Hospital, Shaoxing University, Shaoxing, China

**Keywords:** blood pressure, hypotension, extremely preterm, low perfusion, patent ductus arteriosus

## Abstract

With the development of neonatal medicine, more and more extremely preterm infants have been treated. How to deal with hypotension is a big challenge for neonatologist in the process of diagnosis and treatment. The lack of uniformity in the definition of hypotension, challenges in measuring blood pressure accurately, and insufficient consistency between digital hypotension and hypoperfusion are the primary causes. How to check for hypotension and monitor blood pressure is thoroughly explained in the article. To give neonatologists a resource for the clinical management of hypotension in extremely preterm.

## 1 Background

The extremely preterm population is prone to low systemic blood pressure (BP) (1). According to statistics, 15%–50% of extremely preterm will have hypotension early after birth, and the incidence of hypotension is closely related to gestational age and birth weight (2). It is difficult to do trials of hemodynamic support in extremely preterm (3). Decades of debate have centered on determining the ideal blood pressure range for extremely preterm infants and when to treat hypotension (4). The definition of hypotension, according to experts, can be further defined as follows: a loss of tissue integrity, a loss of function, or a lack of autoregulation of critical organ blood flow (5). Low blood pressure in premature newborns is typically treated by clinicians with the goal of stabilizing or enhancing brain perfusion (6). Though there is growing concern that anti-hypotensive medications may be harmful, there is little evidence to support the idea that they improve outcomes for preterm newborns (7, 8). Clinicians worldwide have differing approaches to managing hypotension in preterm neonates (9–11). This paper describes the monitoring of blood pressure and evaluation of hypotension in extremely preterm.

## 2 Blood pressure monitoring

The method that gives the most accurate readings has the least variability and the lowest likelihood of complications or side effects is the best one for measuring blood pressure. Blood pressure measurements from non-invasive oscillometric or intra-arterial manometric methods have been used to divide previous investigations into two groups (4). In reality, oscillometric techniques are utilized for the majority of neonates in the NICU, and intra-arterial monitoring is only used to assess blood pressure in unstable, severely ill newborns (12). The distinction between invasively transduced and noninvasive oscillometric measures is well-established (13, 14). These variations might not be permanent and change based on the baby's gestational age and the noninvasive device manufacturer.

### 2.1 Noninvasive blood pressure (NIBP) monitoring and precautions

Oscillometric instruments measure the artery's pulsation amplitude. The cuff is inflated above the systolic pressure, and as it progressively deflates, the amplitude of the arterial pulsations increases to a maximum, from which the mean arterial pressure (MAP) is calculated (15). Systolic blood pressure (SBP) and diastolic blood pressure (DBP) are computed using pulse pressure estimates and proprietary computational algorithms from each device vendor. Selecting the appropriate cuff size is the first step in noninvasively measuring a newborn's blood pressure. According to consistent findings, the most accurate blood pressure readings are obtained when the cuff size is chosen such that the ratio of the cuff width to arm circumference is roughly 0.5 (12). An inadequate cuff size may cause an overestimation of blood pressure. The upper arm was shown to be the most precise and least variable place for oscillometric blood pressure measurement in studies looking into measurement location (12). NIBP measurements should be conducted in a peaceful, resting state. Research has indicated that performing up to three measurements improves precision, with the second or third reading exhibiting greater accuracy (16). Additionally, using cuffs frequently can lead to ischemia injury, especially in individuals with delicate skin like preterm newborns (17).

Study has examined the accuracy of a new wearable gadget called Boppli (PyrAmes Inc., Cupertino, CA, USA) that uses machine learning-trained algorithms in conjunction with capacitive sensing to continuously and non-invasively infer blood pressure without the need for external calibration. They showed that the device can be used to treat a variety of diseases in critically ill term and preterm neonates, including severe cardiac illnesses, pre- and post-operative cases, neurological problems, and prematurity (17).

### 2.2 Invasive blood pressure (IBP) monitoring and precautions

IBP readings could not always be reliable, even though they have been regarded as the gold standard. Research has indicated that an infant's results from many measurements may vary (16). An arterial catheter, either centrally implanted through the umbilical artery or peripherally inserted through the radial artery, is used to assess blood pressure as a regular procedure (18). Although it offers direct measurement, there are a lot of issues with this approach. An inaccurate systolic blood pressure reading may result from the use of a tiny diameter catheter in small caliber arteries. Technical factors such as dampening, air bubbles, and zeroing could cause inaccuracies in the measurement of IBP. The most frequent issues with peripheral artery cannulation (PAC) were either the line's inability to bleed back or damping of the trace, which resulted in line removal (19). In addition, a catheter can stay in an artery for a few days and cause major side effects include tissue and nerve damage, bleeding, clotting, and infection (17).

### 2.3 Comparison of two measurement

A study found that there is a significant difference in the 95% limits of agreement between IBP and NIBP, which can cause an overestimation or underestimation of blood pressure. This held for every analyzed subgroup. When comparing invasive SBP and non-invasive SBP, there was a significant bias and wide ranges of agreement; however, when comparing invasive MBP with non-invasive MBP, there was less bias. Moreover, there was little bias for MBP in preterm children who were not given vasoactive medications or in those whose blood pressure remained steady while taking them. Furthermore, they discovered that, in comparison to lower extremities, there was less bias between invasive and non-invasive MBP measures in the upper extremities. Nonetheless, there was a significant bias between the IBP and NIBP groups in preterm infants with hypotension (18). In studies that concentrated mostly on the first five days of birth, nearly all analyses revealed that the oscillometric method produced greater blood pressure readings than the intra-arterial method (20, 21). Neither birth weight nor gestational age at birth showed any clear correlations. Currently, using an in-dwelling intra-arterial catheter to measure blood pressure in neonates is the gold standard method (12). However, we need to depend on NIBP values to screen for BP abnormalities because it is neither practicable nor viable to do IBP monitoring in all ill preterm neonates. Nevertheless, if there are any worries about the hemodynamic state, or the need to start using vasoactive medications, IBP monitoring is essential. Prior to starting vasoactive medications, we should also rely on repeated IBP/NIBP measures in addition to other clinical observations.

### 2.4 Factors affecting the measurement

Research has noted that oscillometric devices are more sensitive to movement and an infant's level of arousal and that their findings can vary more when compared to direct measurements (14, 22). In both extremely and very preterm newborns, Kelsee et al. discovered that blood pressure fluctuation was larger when sleeping prone than when supine (23). The clinical relevance of compromised autonomic regulation in the prone position and its correlation with circulatory instability in preterm newborns throughout the early neonatal period need to be investigated further.

## 3 Blood pressure of extremely preterm

### 3.1 Factors affecting blood pressure

Numerous factors both before and after delivery can impact the blood pressure of infants born extremely prematurely (24). As postnatal age increases, the blood pressure of extremely preterm infants who have positive short-term outcomes rises steadily. African-American race and the completion of a prenatal steroid course were individually associated with higher starting blood pressure values with a slower rate of rise, while the presence of chorioamnionitis was linked to lower starting blood pressure values and a faster rate of rise (4). Delaying the clamping of the chord would cause blood pressure to increase more slowly, which is linked to better hemodynamics (perhaps because of enhanced right ventricular function and vena cava blood flow) (25, 26). According to a study, a much higher percentage of newborns in the group receiving vasopressors had admission temperatures below 36.5°C (1). This implies that blood pressure is affected by temperature. Blood pressure may also be impacted by vasodilation brought on by hypoxia, hypercapnia, neonatal asphyxia, and brain sparing (27, 28). A prior study found that additional factors to consider include an increase in left-to-right shunt through a patent ductus arteriosus or changes in mean airway pressure caused by the development of high-frequency oscillatory ventilation for respiratory distress syndrome (29–31). One-minute Apgar scores and early-onset sepsis have been linked to hypotension, according to another study (32). Attention must also be given to neuroendocrine variables, such as hormones and catecholamines, which have an impact on blood pressure through systemic and pulmonary vascular resistance (33). A higher blood pressure was seen by several researchers in the initial hours after delivery, possibly due to the release of adrenaline during parturition (34). Furthermore, alterations in both the systolic and diastolic blood pressure might be brought on by acute pain (35).

### 3.2 Why are extremely preterm more prone to hypotension?

Extremely preterm babies have a harder time adjusting to birth-related changes than full-term babies do, such as a sudden rise in systemic vascular resistance (SVR) (36). This inability to adjust to elevated SVR can result in increased left ventricular afterload (37), which lowers cardiac output. Research on prematurely born animals has demonstrated that the myocytes in their hearts are smaller than those in their hearts at full term (38). In addition, compared to cardiac terms, the number of binuclear muscle cells is low, the number of proliferating cells is high, and the number of apoptotic cells is low, indicating that preterm muscle cells have not matured. Stress, particularly anemia, and exposure to glucocorticoids speed up myocyte maturation. Early in gestation, the sarcoplasmic reticulum is not well established, and transverse tubules are lacking. The sarcoplasmic reticulum's rate of calcium uptake is likewise decreased in the preterm heart. Poorly contractile material and poorly arranged contractile filaments are found in preterm myocytes. The preterm heart has lower levels of troponin C and I expression, which is linked to a reduced maximum Ca^2+^ activated force. As opposed to being arranged in rows between the myofibrils, mitochondria are dispersed throughout the cytoplasm. Younger fetuses also have less established extracellular matrix. These unfavorable conditions are made worse by hypoxia and acidosis and can impact cardiac output and systemic blood flow (36). In cases of extreme preterm, cardiac dysfunction may also result from perinatal hypoxia and infection (39). The extremely preterm infant's capacity to continue producing cortisol in the event of sustained stress may be limited by the immaturity of the adrenal glands (40, 41). Due to the immaturity of the parasympathetic and sympathetic nervous systems, as well as the aortic arch and carotid sinus's chemoreceptors and baroreceptors, the extremely premature infant may find it challenging to maintain sufficient perfusion pressure. Additionally, the weakened very low birth weight newborn has a higher chance of experiencing reperfusion injury due to the autoregulatory system's malfunction (39). Hypoperfusion is also more likely in cases of extreme preterm due to immature endocrine responses. Acute hypoxemia in full-term infants causes a shift of blood flow to essential organs and is linked to increased levels of vasopressin, glucocorticoids, adrenaline, norepinephrine, and adrenocorticotropin. In newborns with very low birth weights, these reactions become dulled.

### 3.3 What is normal?

Perinatal management of extremely preterm infants has evolved significantly over the past 30 years, particularly with increased use of antenatal steroids and magnesium, both of which influence neonatal blood pressure in the first days of life. Consequently, the relevance of early studies to modern neonates is uncertain (42, 43). The study found that in a typical group of 125 extremely preterm infants born at the border of viability [22^+0^ to 24^+6^ weeks’ gestational age (GA)], there was a significant drop in blood pressure, with a nadir reached about 20 h after birth, regardless of the administration of fluid boluses and cardiovascular active drugs (44). They also observed significant differences in blood pressure patterns among infants born at 22, 23, or 24 weeks gestational age, highlighting the potential effects of immaturity on physiology, even for a difference of only 1–2 weeks. They looked into the connection between heart rate and BP development further and found that BP started to rise about 10 h before heart rate did. Moreover, Batton et al. reported a much earlier nadir in blood pressure, occurring as early as 4–6 h after birth (45). A different study revealed a trend of rising blood pressure over the postnatal period, with SBP showing a greater degree of significance than DBP and MBP  (46). According to all these research, following birth, premature newborns’ blood pressure first decreases and subsequently rises.

The two most widely used metrics in neonatal practice to indicate hypotension during the early transitional period are blood pressure that falls below a mean arterial pressure (MAP) of 30 mm Hg or a MAP with a number lower than the infant's gestational age in weeks. These values will be utilized to define hypotension because research indicates that more than 90% of neonates with gestational ages of 23–26 weeks will have a MAP greater than 30 after the first three days of postnatal life (5). In a study where 1,180 blood pressure readings from 42 extremely preterm newborns were evaluated, doctors found a median (interquartile range) blood pressure of 33 (30–36) mmHg (47). Another study discovered that (i) blood pressure grows gradually with postnatal age (ii) systolic blood pressure increases quicker than diastolic blood pressure, and (iii) the estimated mean blood pressure at birth is approximately 33 mmHg in a cohort of very preterm newborns (4). In a different cohort, the mean blood pressure of infants born between 22^+0^ and 24^+6^ weeks GA was measured at 3 h, 24 h, and 48 h, and it was found to be significantly higher than what was predicted by GA-based methods (44). Vesoulis et al. assessed the mean blood pressure of infants born before 28 weeks gestation to be 33 mm Hg, which aligns with their findings (4). These results contribute to the body of research that questions the validity of estimating postnatal blood pressure in very preterm newborns using GA. These modifications might be associated with prenatal care. Further investigation is required.

## 4 Evaluation of hypotension in extremely preterm

### 4.1 Treat or not?

Hypotension is associated with increased mortality and morbidities including bronchopulmonary dysplasia, intracranial hemorrhage, periventricular leukomalacia, poor neurodevelopmental outcomes, severe retinopathy, and potential for intestinal injury in preterm infants (48, 49). Whether a newborn experiences any negative consequences from a low blood pressure reading alone, without any further indications of impaired end organ perfusion, is a crucial question to answer. According to Alderliesten et al., low MAPs did not cause poor neurodevelopmental outcomes on their own, nor did they consistently correspond with a decline in regional oxygen saturation (50). When evaluating the risk-benefit ratio of treating hypotension in extremely preterm, it is necessary to take into account several factors, including the necessity of therapy, the effectiveness of the medications used to raise blood pressure, the potential benefits of this increase in blood pressure, and any potential adverse effects (51). Sometimes less is more in this vulnerable population. Enhancing cerebral blood flow and organ perfusion are proper objectives. Setting evidence-based goals for the management of newborn hypotension is crucial to prevent the consequences of hypotension on extremely preterm, as well as to reduce the use of hydrocortisone and vasoactive infusion and their related side effects (42). To guide therapy, emphasize the significance of describing the physiology of a hemodynamic disturbance with a thorough examination of myocardial function and its link to the circulatory system and loading circumstances (52).

### 4.2 Evaluation perfusion

The assessment of organ perfusion in relation to systemic blood flow is supported by a comprehensive body of evidence, and maintaining a “normal” blood pressure alone is insufficient to provide optimal perfusion. In extremely preterm infants, the link between blood pressure, systemic blood flow, SVR, and blood flow regulation of different organs during the transition to extrauterine life is complex (53, 54). The assessment of all the systems involved in sustaining organ perfusion, such as oxygenation, hemoglobin level, presence of adrenal sufficiency, respiratory function, cardiac function, renal and hepatic function, and autoregulation of blood flow, is necessary to determine the hemodynamic status of a very low birth weight (VLBW) infant. This is because our current understanding of measuring neonatal blood pressure is limited.Preventing shock should be the main objective rather than avoiding numerical hypotension (50). When there is numerical hypotension, assessment results that indicate decreased organ perfusion (such as oliguria) can help the practitioner decide whether to intervene. Additional clinical signs include abnormal physical examination, tachycardia, metabolic and lactic acidosis, and prolonged capillary refill time (55, 56). Nevertheless, some of these symptoms, especially the decreased urine output, might appear later on in the hypoperfusion (57).

#### 4.2.1 Physical examination

Pallor, acrocyanosis, and cold limbs are initial indications of decreased cardiac output because vasoconstriction mechanisms transfer blood from the periphery to critical organs like the brain, adrenal perfusion, and coronary arteries to compensate for decreased tissue perfusion. Lethargy, agitation, difficulty feeding, hypotonia and even coma are signs of neurological symptoms.

#### 4.2.2 Heart rate

Heart rates in preterm newborns vary, with a 120–160 beats per minute “normal range”. One possible reaction to low cardiac output and an unstable hemodynamic situation is tachycardia, which is the primary compensatory mechanism of newborn cardiac output maintenance (58). To determine the perfusion state of a newborn, it is challenging to use heart rate alone due to the influence of various elements, including temperature, pain, sickness severity, gestational age, and central nervous system function. On the other hand, persistent tachycardia that cannot be attributed to a temperature or pain suggests hypovolemia (39).

#### 4.2.3 Perfusion index (Pi) and plethysmography variability index(PVI)

Peripheral perfusion, superior vena cava (SVC) blood flow, cardiac output, and stroke volume in neonates are all correlated with PI, which is calculated as the percent difference in the infrared signal ratio between pulsatile absorbers (arterial blood flow) and non-pulsatile absorbers (tissue, bone, and organ) (59, 60). By calculating the ratio of the pulsatile to non-pulsatile pulse oximetry signal, peripheral PI assesses systemic perfusion (61). Peripheral vasoconstriction or severe hypovolemia are indicated by low PI, whereas vasodilatation is indicated by high PI. PI is responsive to exogenous vasoactive medications, stroke volume, finger temperature, and sympathetic nervous system tone (pain and agitation) (62). The typical place to monitor is the fingertip (63). In a study including preterm children born before a gestational age of 32 weeks, measurements of PI during echocardiography showed a strong correlation with SVC flow (64). For identifying poor SVC flow within the first 72 h of life, a cutoff value of 0.44 exhibited a negative predictive value of 98.6%. According to David et al., infants that have a poor outcome have a lower Perfusion Index signal with less short-term variability (65). In the first twenty-four hours of life, Alderliesten et al. showed that there was no correlation between the Perfusion Index, regional cerebral oxygenation, and fractional tissue oxygen extraction (66). According to another study, there is no correlation between neonatal left ventricular contractility measures and the PI (63). Thus, extensive prospective research is required.

PVI is a measure of the dynamic change in PI that happens during a full respiratory cycle. It is computed as follows: PVI = [(PImax−PImin)/PImax] × 100] during a time span of about 15 s, which is long enough to include many full respiratory cycles.According to the study, almost all newborns experienced a drop in PVI following shock resolution. The PVI dropped by 11% on average, indicating that volume depletion had a significant role in the infants’ shock. This alteration was particularly prominen in newborns experiencing hypovolemic shock. Another study discovered that 20%[(15, 24)] was the median PVI value (67).

#### 4.2.4 Capillary refill time (CRT)

CRT is a widely used clinical method to evaluate the hemodynamic state of preterm newborns, with the chest being the most typically used region. Newborns are considered to have a normal CRT of less than 4 s, yet this can vary greatly and there isn't always a clear association with SVC flow. A CRT value of more than four seconds could indicate inadequate perfusion (68). When considered as an isolated manifestation, CRT has a limited predictive value and is not a valid indicator for diagnosing shock on its own or evaluating the effectiveness of the newborn's therapy (69, 70).

#### 4.2.5 Urinary output(UO)

Although UO has been used to evaluate the state of organ perfusion, its application to neonates is complicated by their transitional physiology and the ensuing prediuretic phase that is followed by a diuretic phase (39). Furthermore, hours after a hypoperfusion episode, UO, which is measured and quantified in milliliters per kilogram per hour (ml/kg/hour), may not fall into an abnormal range. A comparison of UO to total fluid intake may be more useful in evaluating renal perfusion in an individual because fluid intakes can differ significantly in the first 24 h. Following the baby's first day of life, UO ought to level out and represent their fluid equilibrium. After 24 h of age, a UO of less than 1.5 ml/kg/hour along with further hypoperfusion indicators should be checked into (71). Accurate UO can be challenging to measure in neonates without indwelling urinary catheters, although the use of such catheters carries inherent risks.

#### 4.2.6 Serum lactate

Serum lactate is a tissue perfusion measurement that indicates how severe shock is (lactate concentrations increase with progress). Lactate may accumulate in low perfusion conditions but won't circulate until conditions improve. After baseline measurement, lactate can be tested regularly to monitor progress and evaluate treatment effectiveness. It should be highlighted that the existence of liver problems, inborn errors of metabolism, or medicines can complicate the use of serum lactate concentration as a marker of anaerobic metabolism attributable to hypoperfusion (39).

#### 4.2.7 Blood pressure

The pressure mainly depends on the output and systemic circulatory blockage, which mainly distributes the flow to ensure the normal function of the organs. The decrease of blood pressure is often accompanied by the decrease of output or systemic circulation flow and the imbalance between oxygen supply and demand, which leads to cell hypoxia, metabolic disorder and organ dysfunction (72). The relationship between systemic blood flow and blood pressure is weak during transition (64). As such, the current cutoff values for hypotension have low sensitivity for detecting impaired cardiac output. Furthermore, important details regarding the constituents of blood pressure are lost by utilizing mean blood pressure. The various parts of the blood pressure measurement could help us understand the underlying state of circulation better. Left ventricular contractility is mostly reflected in the systolic component. Therefore, a decrease in preload, an increase in left ventricular afterload, or a decrease in myocardial contractility could be the reason for low systolic pressures. Diastolic blood pressure is crucial in estimating coronary blood flow, it represents both the intravascular blood volume and the resting vascular tone. Reduced preload and/or lower systemic vascular resistance are two possible causes of diastolic hypotension. Considering these individual components may offer deeper insights into the underlying physiology, leading to a more comprehensive understanding suitable for publication (73).

#### 4.2.8 Targeted neonatal echocardiography (TNE)

TNE evaluates cardiovascular physiology and neonatal hemodynamics in the neonatal critical care unit by using full echocardiography to increase diagnostic and therapeutic precision (74). To detect deviations from normal anatomy and characterize the underlying physiology, standard TNE should be considered in any neonate presenting with hypotensive symptoms and clinical indications of a low cardiac output state (especially in extremely preterm during the postnatal transition period). In newborns with low systolic blood pressure, evaluation of the left and right hearts’ morphology and function should be part of the assessment of the heart's performance. Critically ill infants can benefit from TNE (75, 76) to assess cardiac output, which can help with BP interpretation. Estimates of pulmonary and systemic blood flow (SBF) are obtained from the computations of right ventricular output (RVO) and left ventricular output (LVO), respectively; However, measures of RVO and LVO do not directly indicate pulmonary or SBF in the presence of fetal shunts. Due to its role in transitory systemic hypotension, characterization of the hemodynamic relevance of a patent ductus arteriosus (PDA) is crucial.

There is a correlation between intravascular volume and the ratio of the inferior vena cava (IVC) to the aorta (Ao) (77). In euvolemic children, the IVC/Ao ratio was roughly 1.0 in earlier research, and an IVC/Ao ratio of 0.8 was thought to indicate severe dehydration. The mean IVC/Ao ratio in the Kieliszczyk et al. research, which included healthy newborns, was 0.73 (SD = 0.17). According to the study's authors, the typical IVC/Ao ratio for healthy neonates is between 0.51 and 0.96 (based on the group under investigation's 10th and 90th percentiles) (78). It was suggested by Anna et al. that during the first days of life, the IVC/Ao ratio stayed stable. The IVC/Ao ratio on the first day was 0.61 (SD = 0.071) and on the second day, it was 0.62 (SD = 0.061) (79) in newborns whose weight loss at 48 h of birth was less than 8% of BW. These data are from children or full-term newborns, and data from extremely preterm infants require more research.

#### 4.2.9 Transcutaneous Doppler ultrasonography

Compared to standard ultrasonography equipment, transcutaneous Doppler ultrasonography is a continuouswave device that is less costly. This method can be used to assess the blood flow velocity across the aortic and/or pulmonary valves and determine cardiac output. Despite being a relatively simple procedure, this approach yields valuable information; yet, interuser variability is considerable (80).

#### 4.2.10 Electrical cardiometry

To evaluate cardiac output in neonates of different gestational maturities, weights, and body surface areas, electrical cardiometry (EC), sometimes referred to as electrical velocimetry, has been presented as a safe, practical, well-tolerated, non-invasive, continuous approach (62). Using surface adhesive electrodes applied to the neck, scapular region, and thorax, EC operates on the basis of electrical bioimpedance, which is computed based on variations in aortic blood flow (62). Better conductivity is achieved when erythrocytes are arranged unidirectionally parallel to blood flow during forward flow during systole. The blood flow abruptly stops during the diastolic filling stage, causing the erythrocytes to organize haphazardly and increasing resistance and dampening conductivity. The waveform produced by this differential in impedance is used to calculate cardiac output, stroke volume, and blood velocity (62). The stroke volume and cardiac output determined by EC are comparable with echocardiograms with estimated same bias, precision, and tolerable percentage error in newborns, according to the authors’ evidence (81, 82). This establishes the validity of EC. It is significant to highlight that reference values for cardiac function in premature neonates on EC are still being investigated and are not yet clinically established. Additionally, a higher proportion of inaccuracy in EC measures raises concerns for infants receiving invasive mechanical ventilation, especially high frequency ventilation, which is frequently utilized in clinical practice for extremely preterm newborns who are critically ill. The inability of EC to measure intravascular volume or preload presents another drawback in hemodynamic assessment. Consequently, the integration of EC in clinical neonatology is now limited to trending cardiac performance and the hemodynamic impact, rather than relying on absolute values for clinical decision-making.

#### 4.2.11 Near-infrared spectroscopy (NIRS)

NIRS is a noninvasive optical device that offers data on neonatal cerebral hemodynamics and is utilized for continuous bedside monitoring of regional cerebral oxygen saturation (rcSO) (83–85). Oxygenated (HbO2) and deoxygenated (HHb) hemoglobin levels can be measured from the reflected light that sensors pick up when a near-infrared light source is passed through the thin skin and skull of a preterm baby. The ratio of HbO2 to Hb can be calculated using spatial resolution techniques or the multi-distance approach; different manufacturers express this ratio as the tissue oxygenation index (TOI,%) or the regional oxygen saturation (rSO2,%), respectively (86). Newborns can be assessed using specialized flexible mini-sensors or smaller adult sensors that can be put to the skin's surface on the forehead, flank, or periumbilical area, respectively (62). Trends in rSO2 or TOI are assumed to reflect trends in regional cerebral blood flow, assuming that cerebral metabolism remains generally constant, leading to a steady oxygen consumption and venous and capillary blood volume (86). In premature newborns, regional cerebral oxygenation may play a significant role in guiding treatment and preventing cerebral hypoxia (87). Clinical practice now commonly uses cerebral NIRS (88). Based on a large observational study of preterm infants delivered before 32 weeks of gestation, reference values have been published (64). After delivery, RcSO2 levels often rise; the normal range is between 55% and 85%, depending on the gestational age. But according to a recent meta-analysis incorporating data from 2,606 newborns, there wasn't enough information to affirm or refute the idea that cerebral NIRS monitoring improves clinical outcome measures (89). There was no association seen in a recent randomized trial between adverse long-term results and rcSO < 55% or >85% (88). It is challenging to agree on normal values of rcSO in preterm newborns due to the variation in absolute values of rcSO between NIRS devices and sensors as well as between different gestational ages (90). However, there are further restrictions on the NIRS method as well. It assesses tissue oxygen saturation, which is a weighted average of arterial, capillary, and venous blood, in only a portion of the forebrain. Even while NIRS oximetry is relatively accurate, its repeatability is rather limited, with variations of up to 8%, particularly after the sensor is replaced. Clinical decision-making is hampered by this imprecision (86).

Other techniques for determining perfusion in infants born with VLBW are currently being researched. When evaluating the functional impact of perfusion changes in the brain, measures of cerebral function such the electroencephalogram and amplitude-integrated electroencephalogram (aEEG) may be helpful adjuncts (39). Additional aEEG research in extremely preterm infants has looked at the connection between neurodevelopmental outcomes, cardiovascular hemodynamics throughout the postnatal transition phase, and bedside readings. Visible light technology, indicator dilution techniques, and arterial pulse contour analysis are further cutting-edge tools and approaches for measuring cardiac output in preterm newborns (39).

The review states that while each index for assessing perfusion has pros and cons, no one index can provide a thorough assessment of perfusion; instead, many assessment items in the clinic, such as blood pressure, perfusion, oxygenation, and cardiac compensation, can be combined.

## 5 Several common conditions of hypotension

### 5.1 Hypotension of extremely preterm

Preterm newborns are at a higher risk of hemodynamic impairment, making the transition from fetal to postnatal life more challenging (91). The myocardium's immaturity, the pulmonary vascular capacity's natural smaller size, the detrimental cardiovascular consequences of lung disease and associated breathing techniques, and variations in cytokine and/or pharmaceutical responsiveness are among the possible causes (92). The poorly formed contractile mechanisms with disordered myofibrils, an immature calcium handling system, and inadequately compliant collagen make up the preterm myocardium. These factors together result in a myocardium that is less capable of withstanding an abrupt increase in afterload (e.g., following removal of the placenta) and has insufficient reserve to handle decreased preload (93). In addition to low adrenal function, infection, and ultimately hypotension, the delay in the normal decline in PVR caused by lung disease, inability to improve cardiac outputs, and persistence of fetal shunts can all contribute to a maladaptive transition (94, 95).

### 5.2 Persistent pulmonary hypertension (PPHN) of extremely preterm

An infantt with PPHN typically presents as having hypoxemic respiratory failure, perhaps with symptoms of systemic hypotension and transductal right-to-left shunting (difference in pre- and postductal oxygen saturation ≥5%). PPHN has been linked to ventricular failure, both left and right (96). Impaired systolic and diastolic myocardial dysfunction may result from elevated right ventricular (RV) afterload. Deceased right ventricular output, dilatation of the right ventricle, and right-to-left transductal shunting all obstruct left ventricular preload, which lowers left ventricular output. Hypoxia and acidosis can worsen myocardial function. Patients with PPHN should be aware of the possibility of (imminent) ventricular failure because treating systemic hypotension by raising systemic vascular resistance can worsen myocardial function, which can lead to low cardiac output.

### 5.3 PDA

The incidence of PDA inextremely preterm delivery is relatively high, which is related to the immature development of ductal smooth muscle, insensitivity to postnatal oxygen environment stimulation and plasma prostaglandin level (97). Delayed closure of PDA can cause continuous left-to-right shunt of the catheter and increase the volume load of immature muscle cells, resulting in ventricular dysfunction, resulting in reduced systemic circulation flow and low blood pressure (98, 99). The degree of left-to-right shunting through the PDA and ductal steal determines the clinical outcomes. When prematurity is present, an increase in pulmonary blood flow can result in left atrial and ventricular overload and dilatation, respiratory worsening, pulmonary hypertension, pulmonary edema, and left ventricular dysfunction (100). Blood pressure variations resulting from hsPDA can first show up as a fall in diastolic blood pressure in comparison to systolic blood pressure, and eventually both systolic and diastolic blood pressure (62).

### 5.4 Sepsis

Inflammatory response syndrome brought on by sepsis results in peripheral vasodilation and capillary vessel bed leakage. Changes in endothelial function and increased release of proinflammatory cytokines mediate this. The left ventricle is more likely to become hyperdynamic in an effort to raise CO, and there is an initial compensatory rise in heart rate. This results in a clinical picture known as “warm shock,” which is characterized by low diastolic blood pressure and a normal systolic blood pressure. Cardiovascular drugs like norepinephrine, which have strong vasopressor effects, can be used to control this phase. However, in the event of sepsis combined with an elevation in SVR in an attempt to redirect blood to vital organs, myocardial contractility can rapidly deteriorate—a condition known as “cold shock”. As a result, the systolic blood pressure drops (101).

## 6 Conclusion

To sum up, challenges associated with measuring blood pressure accurately in extremely preterm infants include the coexistence of several illnesses, the unpredictability of the transition to extrauterine life, and the challenge of determining organ perfusion. Adequately infused EPT frequently evaluates hypotension based on published blood pressure reference data, but a variety of circumstances make it challenging to decide whether or not to start an intervention for hypotension. Therefore, a thorough and individualised hemodynamic assessment is essential to the prompt diagnosis of cardiovascular failure, the understanding of the underlying pathophysiology, the direction of hemodynamic therapy, the monitoring of the impact of treatments, and the appropriate modification of treatment. The solution to these issues and the aid of computer scientists and biomedical engineers in developing large collaborative networks based on continuous data are large collaborative networks that assist treat the extremely preterm patient more precisely and efficiently than currently possible.
